# Niche Modification by Sulfate-Reducing Bacteria Drives Microbial Community Assembly in Anoxic Marine Sediments

**DOI:** 10.1128/mbio.03535-22

**Published:** 2023-03-29

**Authors:** Qi-Yun Liang, Jin-Yu Zhang, Daliang Ning, Wen-Xing Yu, Guan-Jun Chen, Xuanyu Tao, Jizhong Zhou, Zong-Jun Du, Da-Shuai Mu

**Affiliations:** a Marine College, Shandong University, Weihai, People’s Republic of China; b State Key Laboratory of Microbial Technology, Institute of Microbial Technology, Shandong University, Qingdao, China; c Institute for Environmental Genomics, University of Oklahoma, Norman, Oklahoma, USA; d State Key Joint Laboratory of Environment Simulation and Pollution Control, School of Environment, Tsinghua University, Beijing, China; University of California, Irvine

**Keywords:** sulfate-reducing bacteria, biotic interactions, molecular ecological networks, microbial community assembly, organic matter degradation, sulfate-reducing organisms

## Abstract

Sulfate-reducing bacteria (SRB) are essential functional microbial taxa for degrading organic matter (OM) in anoxic marine environments. However, there are little experimental data regarding how SRB regulates microbial communities. Here, we applied a top-down microbial community management approach by inhibiting SRB to elucidate their contributions to the microbial community during OM degradation. Based on the highly replicated microcosms (*n* = 20) of five different incubation stages, we found that many microbial community properties were influenced after inhibiting SRB, including the composition, structure, network, and community assembly processes. We also found a strong coexistence pattern between SRB and other abundant phylogenetic lineages via positive frequency-dependent selection. The relative abundances of the families *Synergistaceae*, *Peptostreptococcaceae*, *Dethiosulfatibacteraceae*, *Prolixibacteraceae*, *Marinilabiliaceae*, and *Marinifilaceae* were simultaneously suppressed after inhibiting SRB during OM degradation. A close association between SRB and the order *Marinilabiliales* among coexisting taxa was most prominent. They contributed to preserved modules during network successions, were keystone nodes mediating the networked community, and contributed to homogeneous ecological selection. The molybdate tolerance test of the isolated strains of *Marinilabiliales* showed that inhibited SRB (not the inhibitor of SRB itself) triggered a decrease in the relative abundance of *Marinilabiliales*. We also found that inhibiting SRB resulted in reduced pH, which is unsuitable for the growth of most *Marinilabiliales* strains, while the addition of pH buffer (HEPES) in SRB-inhibited treatment microcosms restored the pH and the relative abundances of these bacteria. These data supported that SRB could modify niches to affect species coexistence.

## INTRODUCTION

In anoxic marine sediments, the microbial degradation of organic macromolecules is a complex interspecies process involving hydrolysis, fermentation, and mineralization (1). It has been reported that sulfate reduction can help facilitate the oxidation of up to 50% of the organic matter in marine sediments (2). Sulfate reducers are a phenotypic group composed of sulfate-reducing bacteria (SRB) and sulfate-reducing archaea (SRA), causing some researchers to use the term sulfate-reducing prokaryotes (SRP) or sulfate-reducing microorganisms (SRM) (1). The diversity and abundance of SRB have been relatively high in marine sediments (3, 4), implying the vital importance of SRB during sulfate and carbon cycles in marine sediments.

In anaerobic environments with low redox potential, SRB compete with other anaerobes (e.g., fermentative bacteria, proton-reducing acetogenic bacteria, homo-acetogens, and methanogens) for the available common substrates (5, 6), which are products of the organic matter mineralization process. Beyond this competition, SRB might facilitate the growth of other auxotrophic bacterial taxa via the excretion of metabolic products (e.g., biotin) (7). SRB could also engage in strong cooperative interactions in which energy-transducing metabolic interactions are coupled across anaerobic methane-oxidizing archaea (8). As the dominant microorganisms in marine sediments, the dynamic change of SRB abundance could influence the surrounding abiotic or biotic environments and the entire microbial community (9). However, we require greater knowledge about how community assembly would be influenced when a specific community function is blocked.

Community assembly theory was adapted as a general framework for understanding the structuring of natural communities (10) and is increasingly recognized as a viable framework for unifying ecology (11). Currently, microbial community assembly is suggested to be controlled by two complementary mechanisms, which are deterministic processes based on the niche theory (12, 13) and stochastic processes based on the neutral theory (14–16). Ning (17) developed a reliable tool for quantifying the relative importance of a conceptual community assembly processes framework proposed by Vellend (18). There are numerous studies concerning community assembly in various open environments (19–22) and closed experiments (23–25) based on the microcosm models for bridging the gap between theory and nature. Abundant species with broad functions (26) can exert a large-enough influence on their environment to drive recruitment of new taxa during community succession (27). Therefore, there will be a strong relationship between abundant organisms and deterministic processes during community assembly (28). For the abundant functional SRB, we hypothesize that the combined effects of the SRB could strongly influence their localized environment through niche modification processes during the anaerobic mineralization of organic molecules.

Sulfate reduction can be inhibited by molybdate, a divalent oxyanion analogue of sulfate, competing with and inhibiting sulfate transport and activation (29). Molybdate has been used frequently as a specific inhibitor of SRB in environmental studies (30, 31); therefore, we employed molybdate in microcosms models to illustrate the role of SRB within a microbial community. To reduce covariations due to external environmental variability and to maximize covariations due to interactions (32, 33), we conducted a highly replicated microcosm study (*n* = 20) with homogenized coastal sediments to minimize variability between replicates. In these microcosms, a total of 180 homogenized sediment samples were incubated with a nutrient medium (7) under anoxic conditions. Half of these microcosms were incubated under conditions where sulfate reduction was inhibited by molybdate to elucidate the roles of SRB. The degradation of organic matter was monitored by measuring the concentrations of total organic carbon (TOC), total inorganic carbon (TIC), volatile fatty acids (VFAs), and sulfate. Microbial community composition, assembly, and molecular ecological networks (MENs) were investigated using high-throughput sequencing data of the 16S rRNA genes. Here, we wanted to address two questions during the anaerobic degradation of organic molecules in marine sediments, (i) how SRB plays critical roles within microbial community networks, and (ii) how SRB contributes to microbial community assembly. Our work identifies a previously undocumented dimension of the SRB and offers insight into the ecological properties of this functional microbial group. This targeted inhibition approach can also provide a new framework to study specific functional groups in ecosystems and links between microbial community composition and ecosystem function.

## RESULTS

### Organic matter mineralization is blocked by molybdate.

**FIG 1:**
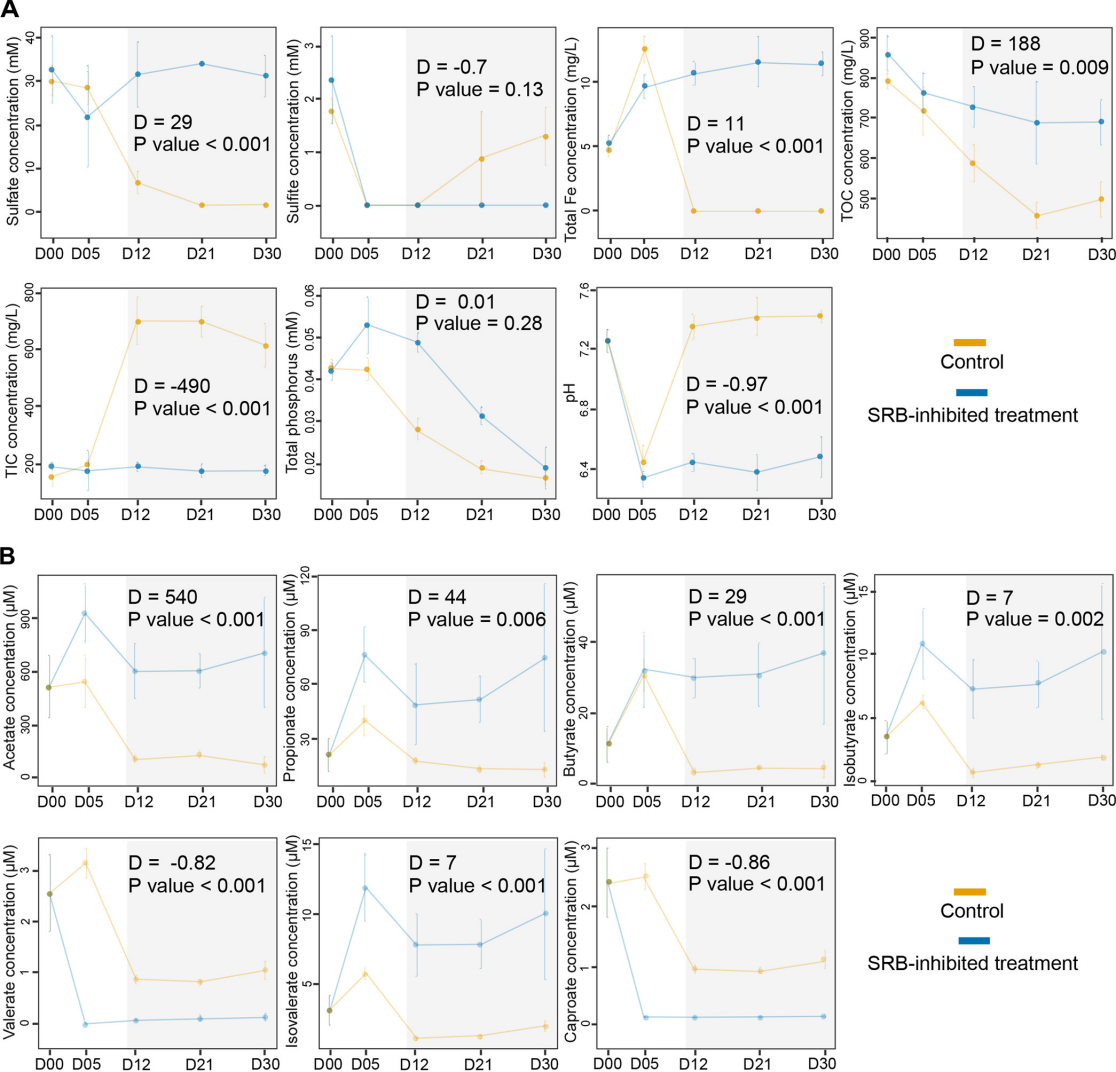
Characterization of physicochemical factors and volatile fatty acids during incubation. (A) Characterization of physicochemical factors. (B) Characterization of volatile fatty acids (VFAs). Orange and blue represent the control and SRB-inhibited treatment groups, respectively. D00, D05, D12, D21, and D30 on the *x* axis represent 0, 5, 12, 21, and 30 days of incubation. Error bars represent the standard deviation. The results of ANOVA with Turkey’s HSD tests from days 12 to 30 are marked in the gray area.

Sulfate concentration sharply decreased from days 5 to 12 in the control group, and the changes from days 5 to 30 showed slight variations in the SRB-inhibited treatment group (Fig. 1A). The changes in sulfate concentration from days 12 to 30 between the control and SRB-inhibited treatment groups displayed significant differences, suggesting that sulfate reduction was fully blocked in the latter. The concentration of total soluble Fe, total organic carbon (TOC), total inorganic carbon (TIC), and pH also showed significant differences between the control and SRB-inhibited treatment groups from days 12 to 30. In contrast, the sulfite concentration and phosphorus did not display significant differences. There were significant differences in TIC from days 12 to 30 between the control and SRB-inhibited treatment groups. All volatile fatty acids (VFAs) showed significant differences during this period of incubation between the control and SRB-inhibited treatment groups (Fig. 1B). The concentrations of primary VFAs (acetate, propionate, and butyrate) were not sharply reduced and maintained a steady state, consistent with the accumulation of VFAs observed by Sørensen et al. (34), showing that the oxidation of VFAs was inhibited.

### Inhibiting SRB altered the bacterial community composition and structure.

**FIG 2:**
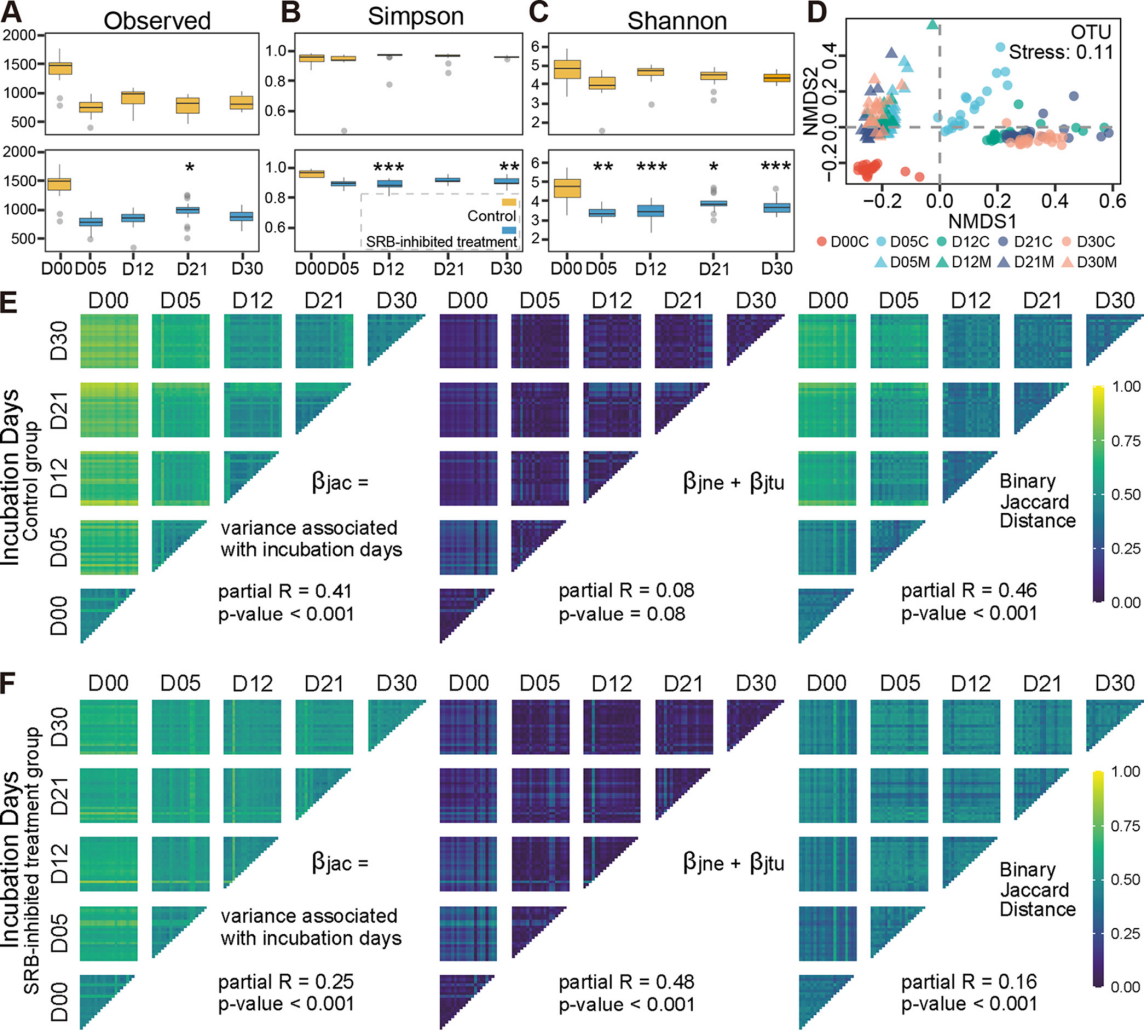
Characterization of bacterial community biodiversity. (A to C) The alpha diversities (observed OTU number [A], Simpson index [B], and Shannon-Wiener index [C]), along with incubation days, both in the control and SRB-inhibited treatment groups, were calculated. Orange and blue represent the control and SRB-inhibited treatment groups, respectively. D00, D05, D12, D21, and D30 on the *x* axis represent 0, 5, 12, 21, and 30 days of incubation. The results of ANOVA with Turkey’s HSD tests between the control and SRB-inhibited treatment groups at the same incubation days are shown; *, 0.01 < *P ≤ *0.05; **, 0.001 < *P ≤ *0.01; and ***, *P ≤ *0.001. (D) Beta diversity of the bacterial community visualized using NMDS ordination based on the binary Jaccard distance. Circle and triangle symbols represent the control and SRB-inhibited treatment groups, respectively. (E and F) Binary Jaccard distance between samples, partitioned by contributions from nestedness (species loss) and turnover (species replacement). The total change (β_jac_) is the sum of changes due to nestedness (β_jne_) and turnover (β_jtu_).

After quality control of high-throughput sequencing data from the 16S rRNA genes, 3,808,147 sequences were generated from 180 samples, ranging from 20,031 to 21,434 sequences per sample. Based on a 97% sequence identity cutoff, 3,337 operational taxonomic units (OTUs) were identified from all samples, ranging from 341 to 1,781 per sample. Alpha diversities decreased significantly from day 0 to day 5 and remained stable for the rest of the incubation (Fig. 2A to C and Fig. S1A to E in the supplemental material). The bacterial community diversity indexes (Shannon-Wiener index and inverse-Simpson index) showed significant differences from days 5 to 30 (Fig. 2C and Fig. S1C). The bacterial communities of the SRB-inhibited treatment group showed lower evenness on days 5 and 12 than the control group (*P < *0.001) and no significant difference from days 21 to 30 (Fig. S1F and G). The structure of the bacterial community was significantly different between the control and SRB-inhibited treatment groups (Fig. 2D and Fig. S1H; Table 1 and Table S1). In detail, the differences in the bacterial community in SRB-inhibited treatment group were smaller than that in the control group (Table 1 and Table S1). Meanwhile, we used a simple metric to count the fraction of the community represented by OTUs that were undetected at any previous time and found that the proportion of detected novel OTUs remained at 8% from days 5 to 12 and then diminished to approximately 2% from days 21 to 30 in the control group, while this proportion remained at about 4% from days 5 to 30 in SRB-inhibited treatment group (Fig. S1I).

**TABLE 1:** Significance tests of the bacterial communities

Comparison	Adonis		ANOSIM		MRPP	
	*F*	*P*	*R*	*P*	δ	*P*
D00C_vs_D05C	19.38	0.001	0.95	0.001	0.49	0.001
D00C_vs_D12C	25.63	0.001	1.00	0.001	0.48	0.001
D00C_vs_D21C	29.72	0.001	1.00	0.001	0.48	0.001
D00C_vs_D30C	34.12	0.001	1.00	0.001	0.46	0.001
D00C_vs_D05M	13.21	0.001	0.88	0.001	0.49	0.001
D00C_vs_D12M	12.60	0.001	0.82	0.001	0.49	0.001
D00C_vs_D21M	11.70	0.001	0.79	0.001	0.49	0.001
D00C_vs_D30M	14.07	0.001	0.93	0.001	0.49	0.001
D05C_vs_D05M	11.99	0.001	0.88	0.001	0.50	0.001
D12C_vs_D12M	20.27	0.001	0.92	0.001	0.49	0.001
D21C_vs_D21M	23.93	0.001	1.00	0.001	0.49	0.001
D30C_vs_D30M	26.95	0.001	1.00	0.001	0.47	0.001

10.1128/mbio.03535-22.1 FIG S1 The rank distribution, biodiversities, and composition analysis of bacterial communities. (A to E) The alpha diversities, along with enrichment incubation days, both in control and SRB-inhibited treatment groups were calculated by using R (version 3.6.3) package microeco. (F) Log-linear rank abundance distributions (RADs) of the top 50 OTUs are shown. RADs perform heavier tails, meaning that the top 50 OTUs usually have relatively high abundance. (G) The average value of the slope (jittered dots represent the slope for each individual replicate) is compared between the control and SRB-inhibited treatment groups. D00, D05, D12, D21, and D30 represent 0, 5, 12, 21, and 30 days of incubation. Orange and blue lines represent the control and SRB-inhibited treatment groups, respectively. (H) The beta diversities of bacterial communities visualized using NMDS ordination based on the binary Jaccard distance (at the family level). Circle and triangle symbols represent the control and SRB-inhibited treatment groups, respectively. (I) Proportion of previously unobserved OTUs. (J and K) The bacterial community composition of abundant taxa at the family level in the control and SRB-inhibited treatment groups, respectively. The outermost two circles list the names of each group and each family. The width of the connection line on the outer ring represents the percentages of bacteria in these groups and correlates with the relative abundance of different family taxa. The top 20 abundant bacterial families are shown, and the remaining families were combined as “other.” The bacterial families are marked by numbers with circles. The gray circles represent shared families between the control and SRB-inhibited treatment groups. The segments in all groups are colored randomly based on the different families. D05, D12, D21, and D30 represent 5, 12, 21, and 30 incubation days and are colored blue, orange, gray, and red, respectively. “C” and “M” represent the control and SRB-inhibited treatment groups, respectively. Significant differences are marked. (ANOVA with Turkey’s HSD tests; *, 0.01 < *P ≤ *0.05; **, 0.001 < *P ≤ *0.01; and ***, *P ≤ *0.001). Download FIG S1, TIF file, 3.5 MB. Copyright © 2023 Liang et al. 2023 Liang et al. https://creativecommons.org/licenses/by/4.0/ This content is distributed under the terms of the Creative Commons Attribution 4.0 International license.

10.1128/mbio.03535-22.7 TABLE S1 Significance tests of the bacterial community structure. Download Table S1, XLSX file, 0.01 MB. Copyright © 2023 Liang et al. 2023 Liang et al. https://creativecommons.org/licenses/by/4.0/ This content is distributed under the terms of the Creative Commons Attribution 4.0 International license.

To understand how much variation in community structure was due to the gain and loss of OTUs, a rigorous analysis of beta diversity was employed, which accounted for the additive contributions of nestedness (species loss) and turnover (species replacement) to the binary Jaccard distance (total beta diversity) calculated between all samples (35). Turnover was a much greater contributor to the observed diversity than nestedness both in the control and SRB-inhibited treatment groups (Fig. 2E and F). Within the SRB-inhibited treatment group, more than 40% of the variance observed was from nestedness, 16% variance was from turnover, and 25% of total Jaccard could be attributed to incubation days (Fig. 2F). Although turnover was high, it was relatively constant between each incubation period. Within the control group, turnover was more variable between samples, and changes to the bacterial parts of the community coincided more with changes in turnover.

The composition of the bacterial community was significantly different between the control and SRB-inhibited treatment groups as revealed by three nonparametric dissimilarity analyses (Table 1). The phylogenetic lineages of abundant taxa between the control and SRB-inhibited treatment groups were significantly different (Fig. S1J and K). In detail, the families *Marinifilaceae*, *Desulfovibrionaceae*, and *Desulfobacteraceae* were abundant taxa only in the control group, while the family *Acidaminococcaceae* was an abundant taxon only in the SRB-inhibited treatment group (Fig. S2A). Based on the results of linear discriminant analysis effect size (LEfSe) (36) analysis, we found that the order *Marinilabiliales* (families *Marinifilaceae*, *Marinilabiliaceae*, and *Prolixibacteraceae*) and some SRB (families *Desulfovibrionaceae* and *Desulfobacteraceae*) showed statistically significant and biologically consistent differences in the control group (Fig. S2B), implying that there would be close associations among them.

10.1128/mbio.03535-22.2 FIG S2 Relative abundance of significantly different abundant taxa between the control and SRB-inhibited treatment groups. (A) Relative abundance and variation of significantly different families between control and SRB-inhibited treatment groups. The graphs in the two lines at the top represent the relative abundance of different families between the control and SRB-inhibited treatment groups. The bottom graph represents the changes of relative abundance in the control and SRB-inhibited treatment groups. Significant differences are marked. (ANOVA with Turkey’s HSD tests; *, 0.01 < *P ≤ *0.05; **, 0.001 < *P ≤ *0.01; and ***, *P ≤ *0.001). (B) Families of statistically significant and biologically consistent differences between the control group and SRB-inhibited treatment groups during incubation. The vertical lines are the cutoff values of linear discriminant analysis (LDA) score based on the LEfSe analysis. D05, D12, D21, and D30 represent 5, 12, 21, and 30 days of incubation and were randomly colored. Download FIG S2, TIF file, 1.4 MB. Copyright © 2023 Liang et al. 2023 Liang et al. https://creativecommons.org/licenses/by/4.0/ This content is distributed under the terms of the Creative Commons Attribution 4.0 International license.

### Inhibiting SRB changed network characters.

The nine molecular ecological networks (MENs) were significantly different from random networks, and all empirical networks showed scale-free and small-world features (Table S2A). The percentage of OTUs for MEN construction was greater under the control than SRB-inhibited treatment group (Table S2A), community members formed looser associations in the latter group, and ecological stochastic processes might be more prevalent in the SRB-inhibited treatment group. Based on the 17 network properties (Table S2A), the succession of MENs under the control and SRB-inhibited treatment groups displayed different trajectories (Fig. S3A and B). In MENs, a module is a group of species that correlate strongly among themselves but little with species in other modules. All MENs both in the control and SRB-inhibited treatment groups were highly modular (Table S2A); meanwhile, the variations of MENs could affect network organizational principles of modularity. Altogether, 27 large modules (modules with ≥5 nodes) accounted for 75 to 88% of the nodes in the MENs in the control group from incubation days 5 to 30, while 26 large modules accounted for 70 to 85% of the nodes in the MENs under SRB-inhibited treatment group during the same incubation stage (Table S2A). The phylogenetic lineages of networked communities (assemblages of microbial taxa detected in the networks) also showed a significant difference between the control and SRB-inhibited treatment groups (Fig. S3C). Based on removing random (5% of the total nodes) and targeted (module hubs) nodes from the networked communities and the relative contribution of a node to the global efficiency, we found that inhibiting SRB could significantly decrease the robustness of MENs and increase vulnerability at incubation days 5, 12, and 30 (Fig. S4A to C). Some significant correlations were detected between partial network stability indices and complexity (Fig. S4D). In detail, node persistence was negatively correlated with the relative modularity, and the robustness (random removal of nodes) displayed a significantly positive correlation with the average degree in the control group (Fig. S4D), meaning that the network stability and complexity in the control group showed a positive correlation, while the robustness (random removal of nodes) was negatively correlated with modularity in MENs of the SRB-inhibited treatment group (Fig. S4D), meaning that the correlation between network stability and complexity in this group was changed to the negative correlation, opposite to that in the control group. We also found that inhibiting SRB increased the node persistence (the percentage of nodes persisting across incubation days) and constancy of empirical networks (Fig. S4E to G), meaning that the compositions of the networked communities in the SRB-inhibited treatment group showed a smaller difference than that in the control group.

10.1128/mbio.03535-22.3 FIG S3 The MENs’ succession, composition, and functional redundancy analysis of networked community. (A and B) Seventeen network topological parameters (listed in Table S2A) were used for nonmetric multidimensional scaling (NMDS) ordination analysis, representing the succession of MENs based on Pearson and Spearman correlation, respectively. Orange circular and blue triangle symbols represent networks in the control and SRB-inhibited treatment groups, respectively. Arrows illustrate the incubation order of the corresponding MENs. (C) The top 20 taxa at the family level are shown, and the remaining families were combined as “others.” The family segments were randomly colored. D00, D05, D12, D21, and D30 represent 0, 5, 12, 21, and 30 days of incubation. “C” and “M” represent the control and SRB-inhibited treatment groups, respectively. Download FIG S3, TIF file, 1.0 MB. Copyright © 2023 Liang et al. 2023 Liang et al. https://creativecommons.org/licenses/by/4.0/ This content is distributed under the terms of the Creative Commons Attribution 4.0 International license.

10.1128/mbio.03535-22.4 FIG S4 Network property analysis of bacterial communities. (A) Robustness measured as the proportion of nodes that remained, with 50% of the nodes randomly removed from each empirical co-occurrence network. (B) Robustness measured as the proportion of nodes remained with 4 module hubs randomly removed from each empirical co-occurrence network. (C) Temporal changes of network vulnerability across incubation days. (D) Pearson correlations between network complexity and stability properties under the control (left) and SRB-inhibited treatment groups (right). Significant (*P ≤ *0.05) correlations are marked, and correlations with *r* values of ≥0.7 are shown in black. (E) Node persistence calculated based on the bacterial community composition in different incubation stages. (F) Number of overlapping nodes in the control and SRB-inhibited treatment groups among different numbers of networks. (G) Each box shows the constancy of all nodes, and Mann-Whitney U test results are displayed. In each panel (except D), the colored orange and blue symbols represent communities under the control and SRB-inhibited treatment groups. D00, D05, D12, D21, and D30 represent 0, 5, 12, 21, and 30 days of incubation. “C” and “M” represent the control and SRB-inhibited treatment groups, respectively. The *r*^2^ and *P* values from linear regressions are shown for the control and SRB-inhibited treatment groups in corresponding colors in panels C and E. Mann-Whitney U test results are shown in panels A, B, and G. *, 0.01 < *P ≤ *0.05; **, 0.001 < *P ≤ *0.01; ***, *P ≤ *0.001. Download FIG S4, TIF file, 1.2 MB. Copyright © 2023 Liang et al. 2023 Liang et al. https://creativecommons.org/licenses/by/4.0/ This content is distributed under the terms of the Creative Commons Attribution 4.0 International license.

10.1128/mbio.03535-22.8 TABLE S2 Network characters. (A) Topological properties of empirical co-occurrence of bacterial communities along enrichment incubation days. (B) Role of individual members within all empirical networks. (C) Role of individual members within preserved modules. Download Table S2, XLSX file, 0.2 MB. Copyright © 2023 Liang et al. 2023 Liang et al. https://creativecommons.org/licenses/by/4.0/ This content is distributed under the terms of the Creative Commons Attribution 4.0 International license.

**FIG 3:**
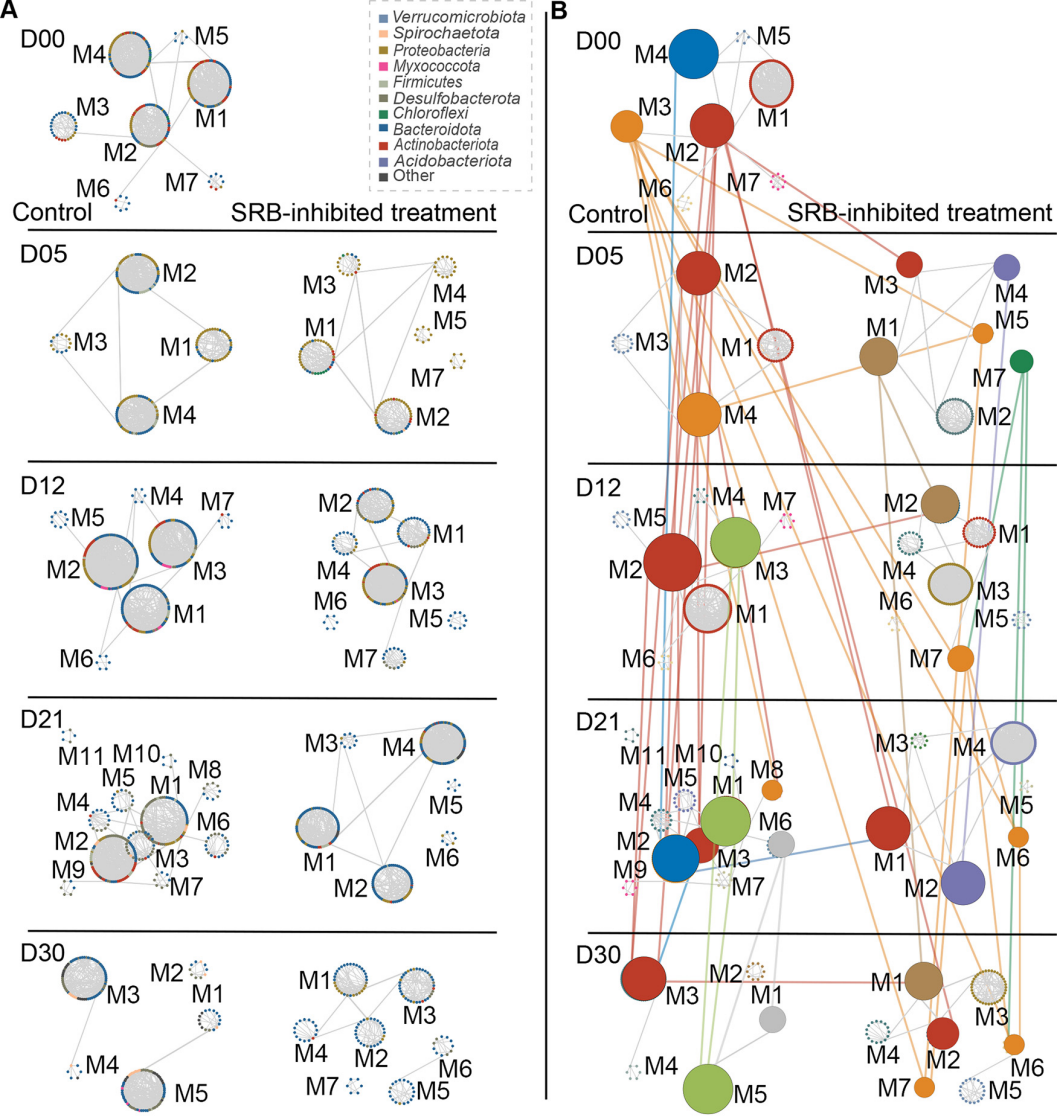
Preserved modules of networks across incubation days. (A) Modules (≥5 nodes) shown in circular layout for all networks. The nodes indicate major taxa and are colored randomly based on the different phyla. The label represents the ID of the module. The left column represents modules in the control group, and the right column represents modules in the SRB-inhibited treatment group. (B) Preserved module pairs are connected and highlighted. Nodes are randomly colored based on the modules. The left column represents modules in the control group, and the right column represents modules in the SRB-inhibited treatment group. The vertical links in the same color indicate that the linked modules were part of the same module clusters. The horizontal links indicate that the linked modules of the control and SRB-inhibited treatment networks at the same enrichment incubation stage were preserved. Note that three clusters of modules (the red, blue, and orange clusters) were consistently preserved over the course of the incubation from D00 (day 0) to D30 (day 30). D00, D05, D12, D21, and D30 represent 0, 5, 12, 21, and 30 days of incubation, respectively. The nodes both in panels A and B only represent different taxa rather than the relative abundance.

An intriguing question is whether inhibiting SRB affects the role of individual members and the principles of network organization. We sought to address this question with two analyses. First, the phylogenetic lineages of keystones (module hubs, network hubs, and connectors) were influenced by inhibiting SRB (Fig. S5A; Table S2B). There were more keystones in the SRB-inhibited treatment group (59 keystones) than the control group (54 keystones), and only 15.3% (15/98) of all keystones were shared between MENs of the control and SRB-inhibited treatment groups (Table S2B). Second, the preserved module pairs were defined as two modules in different networks having a significantly large proportion of shared nodes (37). There were only four preserved module pairs between the control and SRB-inhibited treatment groups, suggesting that inhibiting SRB distinctly altered module identity (Fig. 3). Interestingly, the light-green (M3, M1, and M5 under D12, D21, and D30, respectively) and gray (M6 and M1 under D21 and D30, respectively) clusters of the preserved modules were only detected in the control group (Fig. 3B; Table S2C). In detail, some SRB members made important contributions to the composition of the preserved modules (8/34, 8/23, 4/27, and 5/9, respectively, in M3_M1 of D12C_D21C, M3_M5 of D12C_D30C, M1_M5 of D21C_D30C, and M6_M5 of D21C_D30C [Table S2C]). Meanwhile, some members within the order *Marinilabiliales* made similar contributions to the composition of preserved modules (6/34, 6/23, 8/27, and 1/9, respectively, in M3_M1 of D12C_D21C, M3_M5 of D12C_D30C, M1_M5 of D21C_D30C, and M6_M5 of D21C_D30C [Table S2C]). These results implied that there would be strong associations between SRB and the order *Marinilabiliales*.

10.1128/mbio.03535-22.5 FIG S5 Phylogenetic tree of keystones in all networks and the metabolic reconstruction of the significantly different families. (A) Maximum-likelihood phylogenetic tree of keystone taxa (connectors, module hubs, and network hubs) in all networks under the control and SRB-inhibited treatment groups is displayed at the center. Orange and blue segments in the 1st and 2nd annuli represent the taxa of keystone nodes that appeared in the control and SRB-inhibited treatment networks, respectively. The taxon is colored randomly as the background of tree-leaf labels. (B) The ability of dissimilatory sulfate, assimilatory sulfate, and YcaO-like domain of the significantly different families during incubation between the control and SRB-inhibited treatment groups. Download FIG S5, TIF file, 3.0 MB. Copyright © 2023 Liang et al. 2023 Liang et al. https://creativecommons.org/licenses/by/4.0/ This content is distributed under the terms of the Creative Commons Attribution 4.0 International license.

### The relative importance of community assembly affected by inhibiting SRB.

**FIG 4:**
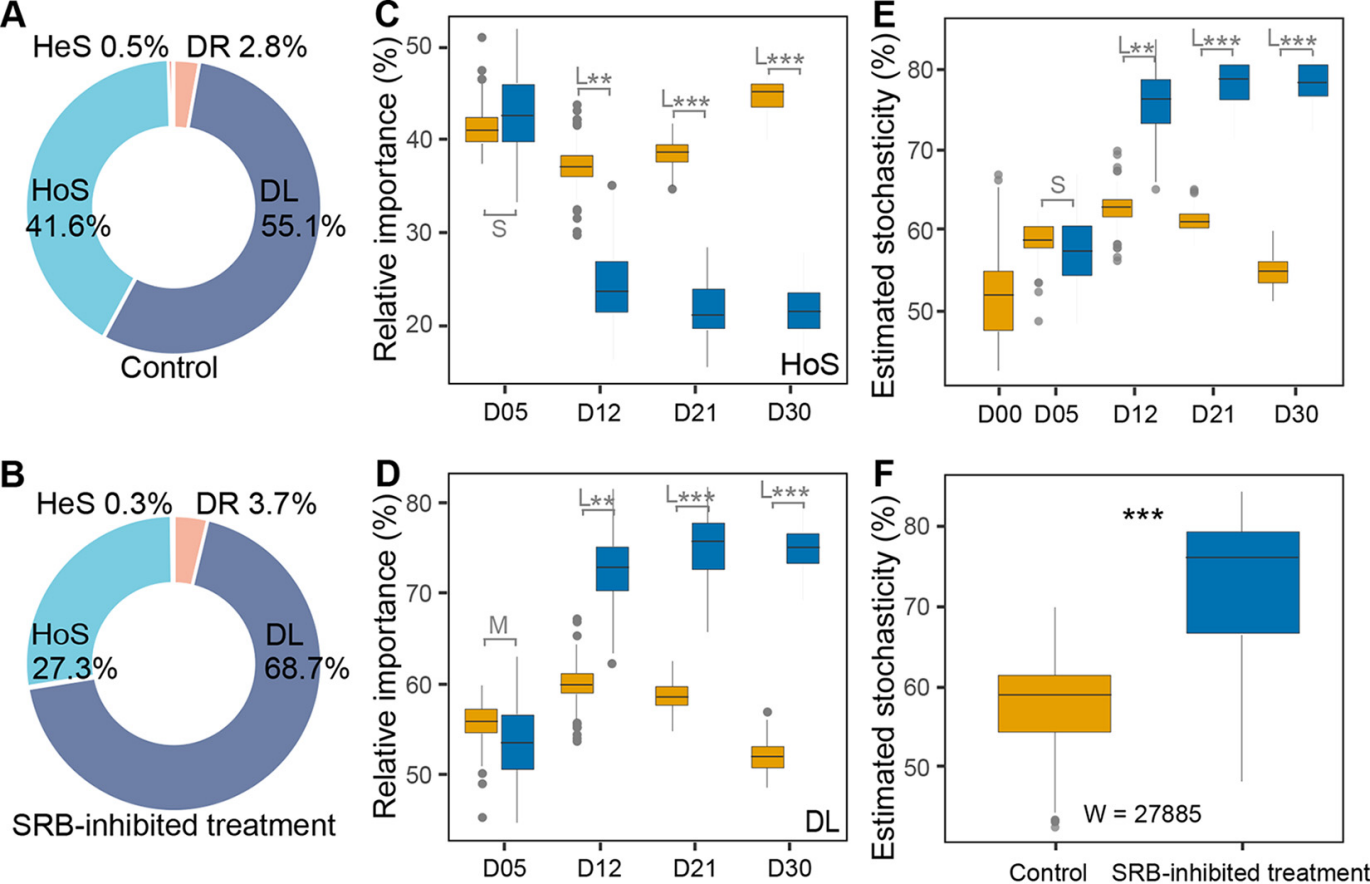
Relative importance of different ecological processes. (A and B) Relative importance of different ecological processes in the control and SRB-inhibited treatment groups, respectively. (DL, dispersal limitation; DR, drift; HoS, homogeneous selection; HeS, heterogeneous selection). (C and D) Changes of homogeneous selection (HoS) and dispersal limitation (DL) in the control (orange box) and SRB-inhibited treatment (blue box) groups. (E and F) Stochasticity estimated in the control and the SRB-inhibited treatment groups across incubation days. In panels C to F, colored orange and blue symbols represent the control and SRB-inhibited treatment groups, respectively. D00, D05, D12, D21, and D30 represent 0, 5, 12, 21, and 30 days of incubation, respectively. L, M, and S represent large (|*d*| > 0.8), medium (0.5 < |*d*| ≤ 0.8), small (0.2 < |*d*| ≤ 0.5), and negligible (|*d*| ≤ 0.2) effect sizes of inhibiting SRB, based on Cohen’s *d* (the mean difference between the control and SRB-inhibited treatment groups divided by pooled standard deviation) in panels C to E. Mann-Whitney U test results are shown in panel F. *, 0.01 < *P ≤ *0.05; **, 0.001 < *P ≤ *0.01; ***, *P ≤ *0.001.

**FIG 5:**
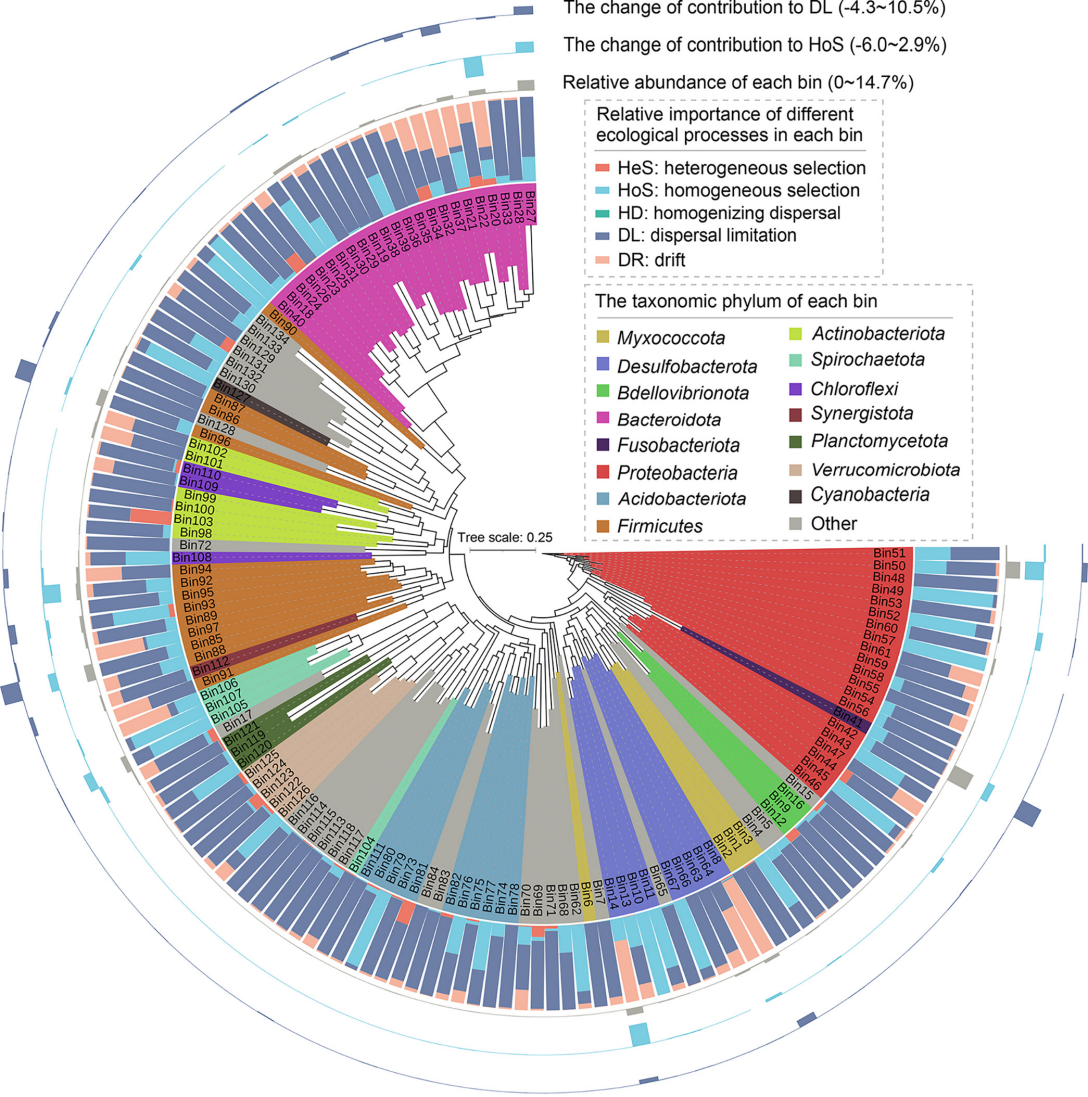
Ecological process across different phylogenetic groups. The maximum-likelihood phylogenetic tree is displayed at the center in a circular layout. All 134 phylogenetic groups are shown in this figure. Bin27, Bin41, Bin50, Bin85, and Bin96 were the five most abundant phylogenetic groups. 1st annulus, relative importance of different ecological processes across different phylogenetic groups; 2nd annulus, relative abundance of each phylogenetic group; 3rd and 4th annuli, the contribution of phylogenetic groups to homogeneous selection (HoS, 3rd) and dispersal limitation (DL, 4th).

The various groups of organisms differ greatly in their responses to environmental changes; some populations are under strong selection, whereas others could exhibit strong drift. Hence, it would be meaningful to consider community assembly processes at the level of individual lineages rather than the entire community (14, 38). We employed the infer community assembly mechanisms by phylogenetic bin-based null model (iCAMP) (17) to detect the effect of inhibiting SRB on bacterial community ecological assembly. Based on the iCAMP results, homogeneous selection (HoS) and dispersal limitation (DL) were found to be the primary ecological processes in community assembly, accounting for up to approximately 90% (Fig. 4A and B). The relative importance of HoS decreased sharply from incubation day 5 to day 12 in the SRB-inhibited treatment group, whereas DL sharply increased (Fig. 4C and D). The bacterial communities in the control group showed a significantly higher ratio of HoS (Cohen’s *d* varied from 4.11 to 10.32, *P < *0.05), but a lower ratio of DL (Cohen’s *d* varied from −4.16 to −10.40, *P < *0.05), supporting that the community assembly was more stochastic in the SRB-inhibited treatment group (Fig. 4E and F). To quantify the relative importance of different ecological processes in each phylogenetic lineage, the 3,337 observed OTUs were divided into 134 individual phylogenetic lineages (Fig. 5). There were 17 primary phylogenetic lineages (>5% relative abundance) dominated by HoS and DL (Fig. 5, 1st and 2nd annuli), suggesting that only a few phylogenetic lineages significantly contributed to ecological processes. In detail, inhibiting SRB decreased the contributions to HoS of Bin14 (*Desulfobacteraceae*), Bin20 (*Marinifilaceae*), and Bin50 (*Vibrionaceae*), while the contributions to DL of Bin27 (*Bacteroidaceae*), Bin41 (*Fusobacteriaceae*), and Bin85 (*Acidaminococcaceae*) were increased by inhibiting SRB, thereby enhancing stochastic processes (Fig. 5, 3rd and 4th annuli).

### Niche modification by SRB promoted species coexistence.

The fact that the relative abundance of different abundant families in the control group decreased by inhibiting SRB surprised us because they are unlikely to be sensitive to molybdate due to the lack of the coding genes of sulfate adenylyl transferase (Sat) and YcaO-like domain (39) (Fig. S5B). The order *Marinilabiliales* among the different abundant families of the control group were prominent due to their closer associations with SRB, including (i) six SRB members and seven *Marinilabiliales* members contributed as keystones within the control group (Table S2B), (ii) the *Marinilabiliales* members made similar contributions to the preserved modules in the control group (Table S2C), and (iii) the relative importance of homogeneous selection in some *Marinilabiliales* members was primary in the control group (Fig. 5).

**FIG 6:**
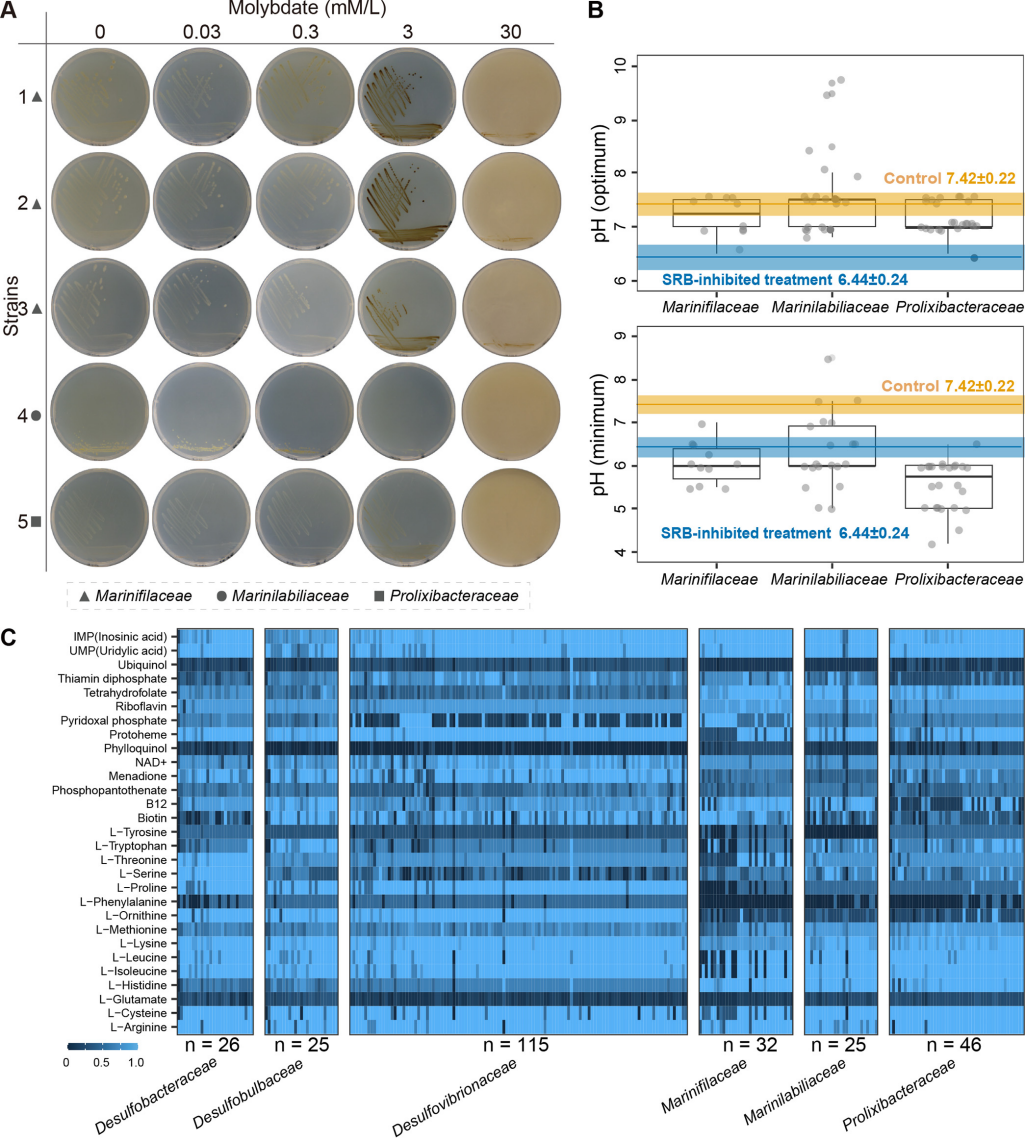
The molybdate tolerance phenotype and growth pH of the order *Marinilabiliales* and metabolic reconstructions. (A) Representative images of the five *Marinilabiliales* strains cultured with molybdate in a series of concentrations (0, 0.03, 0.3, 3, and 30 mM/L) (Table S3B in the supplemental material). Triangle, circle, and square symbols represent the families *Marinifilaceae*, *Marinilabiliaceae*, and *Prolixibacteraceae*, respectively. (B) The two subgraphs display the optimal (top) and minimal (bottom) pH of the order *Marinilabiliales* members validly published according to the International Code of Nomenclature of Prokaryotes (ICNP) (Table S3C). The orange and blue segments represent the variation of pH on incubation days 12 in the control and SRB-inhibited treatment groups, respectively. (C) Metabolic capacities of the family *Desulfobacteraceae* (*n* = 26), *Desulfobulbaceae* (*n* = 25), *Desulfovibrionaceae* (*n* = 115), *Marinifilaceae* (*n* = 32), *Marinilabiliaceae* (*n* = 25), and *Prolixibacteraceae* (*n* = 46) were reconstructed based on the 29 metabolites (15 amino acids, 12 cofactors, and 2 nucleic acids). The blue and dark segments represent the complete and incomplete pathways in metabolic reconstruction, respectively.

To understand why the order *Marinilabiliales* were suppressed in the SRB-inhibited treatment group, we isolated a total of 305 strains from all microcosms based on six different growth media (Table S3A). We then randomly chose five *Marinilabiliales* members from among the isolated strains to test their tolerance to molybdate (Table S3B). The results showed that these strains grew well on marine agar with different concentrations of molybdate (0, 0.03, 0.3, 3, and 30 mM/L) and showed strong tolerance to molybdate (100-fold of concentration in microcosm) in pure culture (Fig. 6A). These results suggested that the inhibited SRB (not the inhibitor of SRB itself) might trigger a decrease in the relative abundance of *Marinilabiliales*.

10.1128/mbio.03535-22.9 TABLE S3 Information of isolated strains and the pH range for growth in the order *Marinilabiliales.* (A) Isolation and classification of bacteria during incubation. (B) Species used in molybdate tolerance and metabolite facilitation studies. (C) pH range of members in the order *Marinilabiliales*. Download Table S3, XLSX file, 0.04 MB. Copyright © 2023 Liang et al. 2023 Liang et al. https://creativecommons.org/licenses/by/4.0/ This content is distributed under the terms of the Creative Commons Attribution 4.0 International license.

Notably, the pH transitioned from weakly acidic to weakly basic during incubation from days 5 to 12 in the control microcosms (Fig. 1A), which is close to the optimal pH of the order *Marinilabiliales* members (Fig. 6B; Table S3C), while in SRB-inhibited treatment microcosms, the pH remained weakly acidic from incubation days 5 to 12 (Fig. 1A), which was close to the minimal pH tolerance of the order *Marinilabiliales* members (Fig. 6B and Table S3C) and unsuitable for the growth of the majority (32/59) of *Marinilabiliales* strains. Thus, we hypothesized that the stable weak base status in microcosms could maintain the relative abundance of the order *Marinilabiliales*. To test this hypothesis, we added the pH buffer (HEPES-free acid) into the SRB-inhibited microcosms. We found that these microcosms restored the pH reduction caused by inhibiting SRB and reinstated weakly basic status in the HEPES addition experiment (Fig. S6A). Meanwhile, the relative abundance of the family *Marinifilaceae* within the order *Marinilabiliales* increased during incubation from days 5 to 12 (Fig. S6B), while the families *Marinilabiliaceae* and *Prolixibacteraceae* within the order *Marinilabiliales* did not significantly increase (Fig. S6B). To some extent, these results supported that the stable weak base status in microcosms could promote the survive of the order *Marinilabiliales*. The loss of biosynthetic genes in bacteria frequently influences their survival in the environment (40). To further assess the biosynthetic ability of certain metabolites, we chose the majority of the abundant taxa in the control and SRB-inhibited treatment groups for comparative genomic analysis and found that many of them were auxotrophic (Fig. 6C; Fig. S6C). Meanwhile, there were complementarities in some metabolites (e.g., l-proline, l-ornithine, l-cysteine, and vitamins B_7_ and B_12_) among SRB and the order *Marinilabiliales*, meaning that SRB could biosynthesize some metabolites, but the order *Marinilabiliales* could not. Therefore, we hypothesized that SRB could provide metabolites (e.g., amino acids and vitamins) to facilitate the growth of auxotrophic *Marinilabiliales* via cross-feeding. To support this assumption, we randomly chose 10 *Marinilabiliales* strains (Table S3B) and found that these strains grew well and were significantly facilitated on the minimal media with various metabolites (e.g., l-proline, l-ornithine, l-cysteine, and vitamins B_7_ and B_12_) (Fig. S6D). The above-described results suggested that niche modification by SRB could modify niches to promote species coexistence, especially with the order *Marinilabiliales*.

10.1128/mbio.03535-22.6 FIG S6 Relative abundance and metabolites facilitation test of the order *Marinilabiliales* and metabolic reconstruction of different abundant families. (A) pH ranges in the control, HEPES addition treatment, and SRB-inhibited treatment groups. D05, D12, and D30 represent 5, 12, and 30 days of incubation. (B) Relative abundance of the order *Marinilabiliales* among control, HEPES addition treatment, and SRB-inhibited treatment groups. The boxes of the control, HEPES addition treatment, and SRB-inhibited treatment are colored orange, gray, and blue, respectively. (C) Metabolic capacities of the significantly different families are reconstructed based on the 29 metabolites (15 amino acids, 12 cofactors, and 2 nucleic acids) (Table S4). The blue and dark segments represent the complete and incomplete pathways in metabolic reconstruction, respectively. (D) Ten *Marinilabiliales* strains (Table S3B) were cultured in the basal media with 0.4% (wt/vol) glucose, supplying seven different metabolites (4 amino acids, 2 vitamins, and 1 cofactor). Triangle, circle, and square symbols represent the families *Marinifilaceae*, *Marinilabiliaceae*, and *Prolixibacteraceae*. The *t* tests are as follows: ns, *P > *0.05; *, 0.01 < *P ≤ *0.05; **, 0.001 < *P ≤ *0.01; and ***, *P ≤ *0.001. Download FIG S6, TIF file, 1.6 MB. Copyright © 2023 Liang et al. 2023 Liang et al. https://creativecommons.org/licenses/by/4.0/ This content is distributed under the terms of the Creative Commons Attribution 4.0 International license.

## DISCUSSION

The degradation of organic matter (OM) in marine sediments is a complex interspecies process involving hydrolysis, fermentation, and mineralization (1). While SRB perform an important process in OM mineralization, how they regulate the microbial community remains unclear. To describe this previously undocumented dimension of SRB, we systematically analyzed the contributions of SRB in the microbial community composition, structure, network, and ecological processes based on comparison with the SRB-inhibited treatment group.

Rapid accumulation of several VFAs demonstrated the primary fermentation of supplemented OM within 5 days of the onset of incubation, which was similar to the degradation of protein and lipid macromolecules in subarctic marine sediment (41). At this stage, pH and TOC concentration were decreased in all incubation microcosms, but not sulfate concentration. After incubating for 5 days, the relative abundance of SRB increased to become abundant in the control microcosms (see Fig. S1J in the supplemental material), coupled with the consumption of sulfate and VFAs (Fig. 1). Meanwhile, the oxidation of VFAs was blocked in SRB-inhibited treatment microcosms (Fig. 1). These results supported that hydrolysis and fermentation are upstream processes during OM biotic degradation (1). SRB contributed to the oxidization of VFAs, shifting the pH from weakly acidic back to weakly basic by removing acidic VFAs and therefore reinstating the pH niche and producing metabolites (e.g., H_2_S), which acted as a strong environmental filter (1) to serve in deterministic ecological processes. Our incubation environments (closed system) were similar to environment pressure (42), reducing the network stability after entering the incubation stage, while the strong environmental filter constructed by SRB slowed the decline of network stability during OM degradation (Fig. S4A and B).

Although the majority of abundant families in the control groups were likely not sensitive to molybdate due to the lack of sulfate adenylyltransferase (Sat) and YcaO-like domain (29, 39) (Fig. S5A), their relative abundances were suppressed through the inhibition of SRB (Fig. S2A). For instance, the *Marinilabiliales* members grew under 30 mM molybdate in pure culture but were significantly suppressed in SRB-inhibited treatment microcosms; this indirect inhibition by molybdate may have possibly been due to a combination of the following. (i) SRB serve as metabolite pool (e.g., amino acids and vitamins) to positively support the growth of these auxotrophs (e.g., *Marinilabiliales*). It has been reported that the majority of microorganisms in nature are auxotrophs, thus relying on external nutrients for growth, including the exchange of amino acids and vitamins (43). These results were similar to those obtained from the free-living bacteria in aquatic systems (44), microbial communities in which syntrophic metabolism of essential enzyme cofactors was found. (ii) SRB have the ability to regulate pH and remediate acidic areas (45). The pH change (acidification) resembles a “public bad,” which is collectively produced members of the population that can kill or inhibit themselves or other members (46). Without the regulation of SRB, the acidic environments generated during the fermentation periods could inhibit the growth of many bacteria belonging to *Marinifilaceae* and *Marinilabiliaceae* (47). Commonly, pH serves as a primary control of microbial communities (48) and microbial interactions among pH-sensitive fermentative species (49). (iii) Sulfides produced by sulfate respiration may regulate the redox potential of the microcosms and thereby act as a factor determining the growth of some anaerobic bacteria (50). Therefore, SRB may apply a great-enough influence on their abiotic and biotic environment to modify their niches. However, additional studies are required to reveal the mechanism of interaction.

Our study revealed the wealth of positive interactions during OM degradation within the control microcosm group, supporting the idea that positive interactions can facilitate other species participating in a “non-zero-sum game” (51, 52). Positive interactions appear to play an important role in supporting microbial community biodiversity (53) and productivity (54, 55). These hypotheses were also supported by this study, in which inhibiting the positive interactions between SRB and other organisms resulted in the repression of OM degradation and a decrease in α-diversity. Many mechanisms could explain the prevalence of positive interactions between SRB and other bacteria in our system. First, given the dynamics of OM degradation, the facilitator might have secreted OM-degrading enzymes that increased intermediate products and overall carbon availability. Second, the facilitator might have excreted incompletely oxidized metabolites that were used by the facilitated strain(s) (56) (e.g., the excretion of VFAs as by-products of incomplete oxidation). Such “overflow metabolism” could allow strains to benefit from the biochemical transformation capabilities of the facilitators (57). Third, the facilitator might exploit new niches by regulating the surrounding microenvironment (e.g., changing pH) to promote resuscitation of facilitated strains from dormancy. Also, facilitated strains might have grown on components from the accumulating dead cells of other bacteria (58).

In experiments with replicate microcosms operated under constant conditions, microbial community composition often follows complex but reproducible trajectories over periods ranging from weeks to months (7, 13, 23, 59). This suggests that taxonomic turnover within functional groups in the absence of obvious environmental variation can be driven by intrinsic and, at least partly, deterministic processes. Even so, every species may be affected by a distinct combination of biotic and abiotic factors that modulate its instantaneous growth rate, even if its metabolic potential overlaps with other members of the community (60). These factors may be frequency dependent and may include a stochastic component, for example, due to drift, mutations, or horizontal gene transfer events (61, 62). In this study, the short period of incubation could not generate an evolutionary process of generating new genetic variation, and the microcosms were made by randomly sampling from homogenized coastal sediments. Thus, following Vellend’s conceptual framework (18), the main community ecology processes in microcosms could be selection and dispersal (Fig. 4A and B). The inhibited SRB coincided with the decrease of homogeneous selection (Fig. 4C) in which homogenous abiotic and biotic environmental conditions lead to more similar structures among communities (14). The sampling from homogenized coastal sediments could not be truly random; thus, the estimated stochasticity was high in all microcosms (>50%) (Fig. 4D). Stochastic processes concern birth/death, speciation/extinction, and immigration, and these could not be detected within closed microcosms based only on the high-throughput sequencing data of the 16S rRNA genes. Therefore, we employed turnover (species replacement) and nestedness (species loss) to represent stochastic processes. Their influence on community dynamics depends on the size and composition of the communities. In the SRB-inhibited treatment group, richness and turnover were lower, while the nestedness was higher (Fig. 2C, E, and F); thus, these results might account for the higher estimated stochasticity in this group (Fig. 4E).

In summary, the present study provides powerful evidence for SRB contributing to microbial ecological services. Our analysis shows that SRB, acting as one of the most active prokaryotic populations in the mineralization of VFAs, diversified community structure during succession, enhanced network stability, contributed to the preservation of network modules, promoted species coexistence with the order *Marinilabiliales*, and significantly contributed to homogeneous selection in community assembly.

## MATERIALS AND METHODS

### Marine sediment sampling and incubation.

All marine sediment samples were collected from the intertidal zone of Weihai, China (37°31′33″N, 122°1′47″E) on 29 August 2018. Sediment from the depths of 5 to 10 cm was collected, placed in 1,000-mL sterile plastic bags, kept cold within an ice box, and immediately (within 4 h) processed for incubation. The incubation medium was referenced from the work of Mu et al. (7) and then modified consisting of the following in natural seawater: 0.1% NH_4_Cl, 0.2% CH_3_COONa, 0.02% MgSO_4_·7H_2_O, 0.02% yeast extract, 0.02% peptone, 0.1% EDTA, and 0.125% sodium pyruvate. The pH of the medium was adjusted to 7.0 and then autoclaved. Ten percent (wt/vol) NaHCO_3_ solution was filtered, and a 2% (wt/vol) KH_2_PO_4_ solution was autoclaved. Each solution was added to the autoclaved media (10 mL per L). The above description served as the incubation medium for the control group, while that of the SRB-inhibited treatment group was amended with Na_2_MoO_4_ as a sulfate respiratory inhibitor at 3 mM/L (final concentration). Twenty-five grams of sediment was added to 250-mL sealed glass bottles, which were then filled with an approximate amount of incubation medium. Then, all bottles were stored at 25°C for 0, 5, 12, 21, and 30 days and shaken twice daily. The control and SRB-inhibited treatment groups both had 20 replicates for each incubation time point. To test the hypothesis that the stable weak base status in microcosms could maintain the relative abundance of the order *Marinilabiliales*, we recollected marine sediments at the same sampling site on 9 May 2022. The microcosms (*n* = 3) in the control and SRB-inhibited treatment groups were prepared following the process of the above-described highly replicated microcosms. Meanwhile, we added HEPES-free acid (4.7 g/L) into the SRB-inhibited microcosms as the pH-restored treatment group (*n* = 3). Then, a total of 27 bottles were incubated at 25°C for 5, 12, and 30 days and shaken twice daily. In the end, all samples were collected and stored at −80°C for 16S rRNA high-throughput sequencing.

### Measurement of physical-chemical factors and SCFAs.

We randomly selected 6 samples from the 20 replicates at each incubation time point for measurement of physical-chemical factors and then randomly chose 4 of those 6 samples for measuring short-chain fatty acids (SCFAs). The supernatant was collected after centrifugation (12,000 rpm for 5 min, 4°C) and stored at −80°C. The concentrations of phosphorus, Fe, sulfate, sulfite, total organic carbon (TOC), and total inorganic carbon (TIC) were measured at the Scientific Instruments Sharing Platform, Third Institute of Oceanography, Ministry of Natural Resources, Xiamen, China (http://www.tio-sisp.net/JMISP/), using standard testing methods. The contents of SCFAs were detected at Shanghai Applied Protein Technology Co., Ltd., China (http://www.aptbiotech.com/), and the targeted metabolite profiling analysis procedure followed those of Wu et al. (63).

### DNA extraction, 16S rRNA gene sequencing, and analysis.

Extraction and purification of microbial community genomic DNA from samples were carried out using the E.Z.N.A. soil DNA kit (Omega Bio-tek, Norcross, GA, USA) according to the manufacturer’s protocols. The V3 and V4 regions of the 16S rRNA genes were amplified using general primers (338F/806R). Sequencing was performed on the Illumina MiSeq PE300 platform (Illumina, San Diego, USA) according to the standard protocols by Shanghai Majorbio Bio-pharm Technology Co. Ltd. (Shanghai, China). Paired-end reads were merged by FLASH version 1.2.11 (64). Altogether, we obtained 4,072,354 high-quality reads (average length, 419 bp) from 180 samples, and the lowest number of reads among the samples, 21,530, was chosen to rarefy data sets for all community comparisons. Operational taxonomic units (OTUs) with 97% similarity cutoff were clustered using UPARSE version 7.0 (65), and chimeric sequences were identified and removed. The taxonomy of each OTU representative sequence was analyzed by RDP Classifier version 2.11 (66) against the Silva version 138 16S rRNA database (67) using a confidence threshold of 0.7.

Alpha diversity metrics were calculated using the R package microeco (68), and the significance of differences in alpha diversities was tested through analysis of variance (ANOVA) with Tukey’s honestly significant difference (HSD) test. Nonmetric multidimensional scaling (NMDS) analysis was performed using R package vegan (69) based on Bray-Curtis dissimilarities. We also employed nonparametric multivariate statistical analysis (Adonis, analysis of similarity [ANOSIM] and multiresponse permutation procedure [MRPP]) based on Bray-Curtis distance to assess whether the bacterial community compositions were different. Rank abundance distributions (RADs) for each sample were calculated using R package RADanalysis (70). The visualization of bacterial community composition was created by Circos (71) online (http://circos.ca/). The differential abundance test of microbial community across the control and the SRB-inhibited treatment groups was analyzed based on the LEfSe (36). To further understand the potential interaction between SRM and differential taxa, we converged differential families in the control group based on the two conditions of whether differential families were abundant taxa and detected on at least two incubation days. To account for the amount of temporal variation in bacterial community structure, the nestedness (species loss) and turnover (species replacement) were employed to partition the beta diversity by using the R package betapart (72).

### Genome metabolic reconstruction.

Twenty-nine significant different families were selected based on OTU abundance. We confirmed 3,596 genomes according to the Genome Taxonomy Database (GTDB; https://gtdb.ecogenomic.org) (73) on the basis of the taxonomic information at the family level of the abundant taxa in the control and SRB-inhibited groups (see Fig. S2A in the supplemental material) and by filtering with completeness ≥80% and contamination ≤5% (Table S4). We then downloaded these confirmed genomes from the NCBI database (https://pubmed.ncbi.nlm.nih.gov/) for downstream analysis. The protein-coding gene prediction for the downloaded genomes was performed by prodigal version 2.6.3 (https://github.com/hyattpd/Prodigal) (74). We employed KofamKOALA (75) (https://www.genome.jp/tools/kofamkoala/) for functional annotation of protein-coding genes. The metabolic pathways of 29 metabolites (including 15 amino acids, 12 cofactors, and 2 nucleic acids) and sulfate reduction were analyzed by KEGG Mapper (76). Meanwhile, sulfate adenylyltransferase (Sat) and YcaO-like domain were employed to confirm whether differential taxa were sensitive to molybdate (29, 39).

10.1128/mbio.03535-22.10 TABLE S4 List of comparative genomic analyses. Download Table S4, XLSX file, 0.8 MB. Copyright © 2023 Liang et al. 2023 Liang et al. https://creativecommons.org/licenses/by/4.0/ This content is distributed under the terms of the Creative Commons Attribution 4.0 International license.

### Network construction and analysis.

**(i) MEN construction.** To investigate the dynamics of microbial community patterns over the course of the incubation, we employed the Molecular Ecological Network Analyses pipeline (MENAP; http://ieg4.rccc.ou.edu/mena/) following the random matrix theory (RMT) approach (77, 78). MENs were constructed with the following settings. Only OTUs present in at least 11 of the 20 samples for each time point were included for Pearson correlation calculation. If OTU abundances had missing data, the blanks were filled with 0.01. OTU abundances were log transformed, calculated by decreasing cutoff from the top; we set the parameter of “scan speed” as “Regress GOE and Poisson” during MEN construction in MENAP, and the similarity correlation cutoff threshold was 0.80. We applied iDIRECT (79) to remove spurious indirect links in the original MENs (https://github.com/nxiao6gt/iDIRECT).

**(ii) Network characterization.** Modules were detected using the greedy modularity optimization method, and various network topological properties were calculated to characterize the topological structure of the MENs by using R packages (igraph, omnivor, and brainGraph), including nodes, links, power-law fitting of node degrees, average degree (avgK), average clustering coefficient (avgCC), average path distance (APD), graph density (GD), modularity, nestedness, relative modularity (RM), and relative nestedness (RN). To classify the nodes based on the topological roles that they play in the network, the connectivity of each node was determined based on its within-module connectivity (*Z_i_*) and among-module connectivity (*P_i_*) (80). We followed criteria used in previous studies and identified four categories (33, 37, 78), module hubs (*Z_i_* ≥ 2.5, *P_i_* < 0.62), network hubs (*Z_i_* ≥ 2.5, *P_i_* ≥ 0.62), connectors (*Z_i_* < 2.5, *P_i_* ≥ 0.62), and peripherals (*Z_i_* < 2.5, *P_i_* < 0.62). Module hubs, connectors, and network hubs are referred to as keystone nodes (37). We applied IQ-TREE version 1.6.12 (81) for phylogenetic analysis of keystone nodes, and the visualization was created on iTOL (82) (https://itol.embl.de/).

**(iii) Network comparison.** To evaluate the differences between MENs, we performed a NDMS analysis based on 17 topological network indices calculated for each empirical MEN. We fitted a linear model between each network index and incubation time to understand how each network property varied over incubation time. We employed Fisher’s exact test to identify preserved module pairs (i) under control and treatment over time, and (ii) between control and treatment at the same time point. The detection of preserved module pairs was calculated following descriptions by Yuan et al. (37).

**(iv) Network stability.** To evaluate the robustness of MENs, we simulated random (5% of the total nodes) or targeted (module hubs) species removal where a certain proportion of nodes were removed (83, 84). To test the effects of species removal on the remaining species, we calculated the abundance-weighted mean interaction strength (wMIS) of the node, following the description by Yuan et al. (37). To measure the relative contribution of a node to the global efficiency, we calculated the vulnerability of each node (78). We then followed the method introduced by Yuan et al. (37) and calculated the node constancy, node persistence, and compositional stability.

**(v) Spearman correlation for network construction.** To explore whether different correlation methods impacted the construction of MENs, we also used Spearman correlation to construct MENs and applied iDIRECT (79) to remove spurious indirect links in the original MENs based on Spearman correlation. We show the topological properties of Spearman correlation-based MENs in Table S2A to certify that the successional trend of MENs derived from Pearson and Spearman correlations over incubation days is consistent. All presented detailed analyses of the MENs are based on Pearson correlation.

**(vi) Random networks construction.** To confirm that the observed MEN topology represents nonrandom assemblies of bacterial communities, 1,000 random networks were generated and compared with the nine empirical MENs. We calculated the topological properties of the random networks, which include average path distance (APD), average clustering coefficient (avgCC), modularity, and nestedness.

### Microbial community assembly analysis.

The infer community assembly mechanisms by phylogenetic bin-based null model (iCAMP) was used to investigate the assembly mechanisms of different microbial groups (https://github.com/DaliangNing/iCAMP1) (17). By using iCAMP, five assembly mechanisms of different microorganism phylogenetic groups (called bins) were identified, including homogeneous selection (HoS), heterogeneous selection (HeS), dispersal limitation (DL), homogenizing dispersal (HD), and drift (DR), which were explained in detail in a previous study by Zhou and Ning (14). Furthermore, the molybdate-induced changes in HoS and DL were investigated in our study, and a change was defined as a positive value if the relative contribution of HoS or DL was higher under sulfate reduction-inhibited conditions than under control. We also employed IQ-TREE version 1.6.12 (81) for phylogenetic analysis of bins, and the visualization was created on iTOL (82) (https://itol.embl.de/).

### The isolation and classification of bacteria during incubation.

Serial dilutions of incubation samples were spread-plated onto various rich media (listed below). Plates were then incubated at 28°C for 7 days. All experiments were performed under aerobic and anaerobic conditions, respectively. The following growth media were used in this study: marine agar (F) supplemented with 1 g/L yeast extract, 5 g/L tryptone, 1 g/L beef extract, and 0.01 g/L FePO_4_·4H_2_O; nutrient agar (J) supplemented with 3 g/L beef extract and 5 g/L tryptone; modified marine agar (L) supplemented with 1 g/L yeast extract, 5 g/L tryptone, 0.08 g/L KBr, 0.057 g/L SrCl_2_, 0.022 g/L H_3_BO_3_, 4 mg/L Na_2_SiO_3_, 0.02 g/L Na_2_HPO_4_, 1.6 mg/L NaNO_3_, and 2.4 mg/L NaF; Trypticase soy yeast extract (X) supplemented with 30 g/L Trypticase soy broth and 3 g/L yeast extract; modified marine agar (Y) supplemented with 1 g/L yeast extract, 5 g/L tryptone, 0.08 g/L KBr, 0.034 g/L SrCl_2_, 0.022 g/L H_3_BO_3_, 4 mg/L Na_2_SiO_3_, 8 mg/L Na_2_HPO_4_, 3.24 g/L Na_2_SO_4_, 2.4 mg/L NaF, 1.6 mg/L NH_4_NO_3_, 8.8 g/L MgCl_2_, 8.8 g/L CaCl_2_, 0.16 g/L Na_2_CO_3_, 0.55 g/L KCl, and 0.1 g/L FeC_6_H_5_O_7_; and modified marine agar (Z) supplemented with 1 g/L yeast extract, 5 g/L tryptone, 0.034 g/L SrCl_2_, 0.022 g/L H_3_BO_3_, 4 mg/L Na_2_SiO_3_, 8 mg/L Na_2_HPO_4_, 2.4 mg/L NaF, 1.6 mg/L NH_4_NO_3_, 0.08 g/L KCl, and 0.1 g/L FeC_6_H_5_O_7_.

### Molybdate tolerance and metabolite facilitation testing.

We randomly chose five *Marinilabiliales* strains for molybdate tolerance testing (Table S3B). The medium was marine agar with Wolin's vitamin solution (https://www.dsmz.de/; DSMZ medium 141) and different molybdate concentration series (0, 0.03, 0.3, 3, and 30 mM/L), and all plates were incubated at 28°C for 48 to 72 h in anaerobic packs. We randomly chose 10 *Marinilabiliales* strains for metabolite facilitation testing (Table S3B). The minimal medium of metabolites (5 amino acids, 2 vitamins, and 1 cofactor) facilitation testing was modified marine agar (without yeast exact and tryptone) using artificial seawater (MgCl_2_·6H_2_O 4.83 g/L, MgSO_4_·7H_2_O 6.66 g/L, CaCl_2_ 1.15 g/L, NaHCO_3_ 0.2 g/L, KCl 0.72 g/L, and NaCl 30 g/L) amended with 0.4% (wt/vol) glucose as the sole carbon source. The concentrations of vitamins followed the description of Wolin’s vitamin solution (DSMZ medium 141). The concentrations of amino acids and menadione were 1 g/L and 0.5 mg/L, respectively. Liquid cultures were followed by passaging 2 μL of the suspension (optical density at 600 nm [OD_600_], 0.2) into 200 μL of fresh media (minimal medium with the metabolites; parallel experiments = 5) in 96-well plates. All 96-well plates were incubated at 28°C for 54 h under aerobic conditions.

### Data availability.

The 16S rRNA gene data sets generated during this study have been deposited in the Sequence Read Archive under accession no. SRP364228 for 207 samples. The list of 207 runs under SRP364228, R scripts, and raw data is available on GitHub at https://github.com/2015qyliang/InhibitingSRB_molybdate.
